# A Single Nucleotide Polymorphism in *Tetherin* Promotes Retrovirus Restriction *In Vivo*

**DOI:** 10.1371/journal.ppat.1002596

**Published:** 2012-03-22

**Authors:** Bradley S. Barrett, Diana S. Smith, Sam X. Li, Kejun Guo, Kim J. Hasenkrug, Mario L. Santiago

**Affiliations:** 1 Department of Medicine, University of Colorado Denver, Aurora, Colorado, United States of America; 2 Department of Microbiology, University of Colorado Denver, Aurora, Colorado, United States of America; 3 Rocky Mountain Laboratories, National Institutes for Allergy and Infectious Diseases, National Institutes of Health (NIH), Hamilton, Montana, United States of America; 4 Department of Immunology, University of Colorado Denver, Aurora, Colorado, United States of America; Duke University Medical Center, United States of America

## Abstract

Tetherin is a membrane protein of unusual topology expressed from rodents to humans that accumulates enveloped virus particles on the surface of infected cells. However, whether this ‘tethering’ activity promotes or restricts retroviral spread during acute retrovirus infection *in vivo* is controversial. We report here the identification of a single nucleotide polymorphism in the *Tetherin* gene of NZW/LacJ (NZW) mice that mutated the canonical ATG start site to GTG. Translation of NZW Tetherin from downstream ATGs deleted a conserved dual-tyrosine endosomal sorting motif, resulting in higher cell surface expression and more potent inhibition of Friend retrovirus release compared to C57BL/6 (B6) Tetherin *in vitro*. Analysis of (B6×NZW)F_1_ hybrid mice revealed that increased Tetherin cell surface expression in NZW mice is a recessive trait *in vivo*. Using a classical genetic backcrossing approach, NZW Tetherin expression strongly correlated with decreased Friend retrovirus replication and pathogenesis. However, the protective effect of NZW Tetherin was not observed in the context of B6 Apobec3/*Rfv3* resistance. These findings identify the first functional *Tetherin* polymorphism within a mammalian host, demonstrate that Tetherin cell surface expression is a key parameter for retroviral restriction, and suggest the existence of a restriction factor hierarchy to counteract pathogenic retrovirus infections *in vivo*.

## Author Summary

Significant portions of the human and mouse genomes are comprised of retroviral sequences, revealing the long history of conflict between mammalian hosts and retroviruses that led to the evolution of host restriction factors. Nucleotide mutations in restriction factor genes provide a glimpse of this ongoing evolutionary process, but studies that directly probe the impact of restriction factor mutations during retrovirus infection are limited. In this study, we identified a single nucleotide mutation in the Tetherin host restriction gene that resulted in retention of Tetherin on the cell surface. In cell culture, Tetherin accumulates virions on the infected cell surface and prevents virion release, but some studies suggested that Tetherin might facilitate cell-to-cell virus spread. Our studies reveal that the Tetherin polymorphism inhibits retrovirus replication and disease. Thus, increased Tetherin cell surface expression enhanced the antiretroviral function of Tetherin. These results could have important implications in harnessing the biology of Tetherin for controlling pathogenic retroviruses such as HIV-1.

## Introduction

In order to infect and persist in the host, retroviruses such as HIV-1 encode proteins that counteract innate resistance genes that are also referred to as “restriction factors”. Host restriction factors have the potential to directly interfere with specific steps of the retrovirus life cycle and have been the subject of intense study in the last decade. In this regard, mechanistic studies on how the HIV-1 Vpu protein promotes virion release *in vitro* resulted in the discovery of the long-sought ‘Tetherin’ molecule [1]–[2]. Tetherin (also known as BST-2, CD317, HM1.24 and PDCA-1) is a homodimeric protein containing an N-terminal transmembrane and C-terminal glycophosphatidyl inositol anchor [3] that ‘tethers’ virions on the surface of the infected cells, resulting in extensive virion aggregation [1]–[2], [4]–[5].

The impact of Tetherin-mediated virion aggregation on retroviral spread is controversial. In pandemic HIV-1 strains lacking the human Tetherin antagonist Vpu, surface-tethered virions associate with the virological synapse, but this interaction has been reported to both inhibit [6]–[7] and promote [8] cell-to-cell virus spread. Human T-Lymphotropic Virus type I [9] and Feline Leukemia virus [10] were also suggested to utilize human and feline Tetherin, respectively, for cell-to-cell spread *in vitro*. In contrast, Tetherin-driven retrovirus evolution in the SIV or SHIV lentivirus infection models [11]–[12] suggested that Tetherin was an antiretroviral factor. Resolving these opposing views may require pathogenic retrovirus infection studies that isolate the *Tetherin* gene *in vivo*.

Recently, a study in *Tetherin* deficient mice demonstrated that Tetherin restricts Moloney Murine Leukemia Virus (MLV) and a pathogenic MLV complex known as LP-BM5 *in vivo* [13]. In that study, Interferon-α (IFN-α) induction through poly(I:C) treatment was required to unmask the activity of Tetherin in newborn mice infected with Moloney MLV. Moreover, the effect of Tetherin on LP-BM5 infection of adult mice was not observed until after 8 weeks post-infection, during the chronic stage and when adaptive immune responses had already developed. In contrast, studies on mice deficient with another restriction factor, Apobec3, revealed a significant impact on retrovirus replication and pathogenesis during the first week of retrovirus infection in both newborn [14]–[15] and adult immunocompetent mice [16]–[18]. Thus, in contrast to Apobec3, the impact of Tetherin during acute retrovirus infection appeared to be minimal, suggesting that Tetherin may not be a potent innate restriction factor *in vivo*.

Comparison of *Tetherin* gene sequences from various mammalian hosts revealed high levels of positive selection in Tetherin, likely reflecting the long-standing genetic conflict between retroviruses and mammalian hosts [19]–[20]. We therefore analyzed *Tetherin* gene sequences from catalogued inbred mouse strain genomes, and report here the identification of a single nucleotide polymorphism (SNP) in *Tetherin* that significantly increased its ability to inhibit retroviral replication and pathogenesis *in vivo*. Due to the nature of this unique SNP, the results revealed that Tetherin cell surface expression is critical for retroviral control. Finally, the classical backcrossing approach highlighted a specific genetic context that revealed potent Tetherin activity during acute retroviral infection *in vivo*.

## Results

### A single nucleotide polymorphism in NZW/LacJ mice results in translation of a truncated Tetherin protein

**Figure 1:**
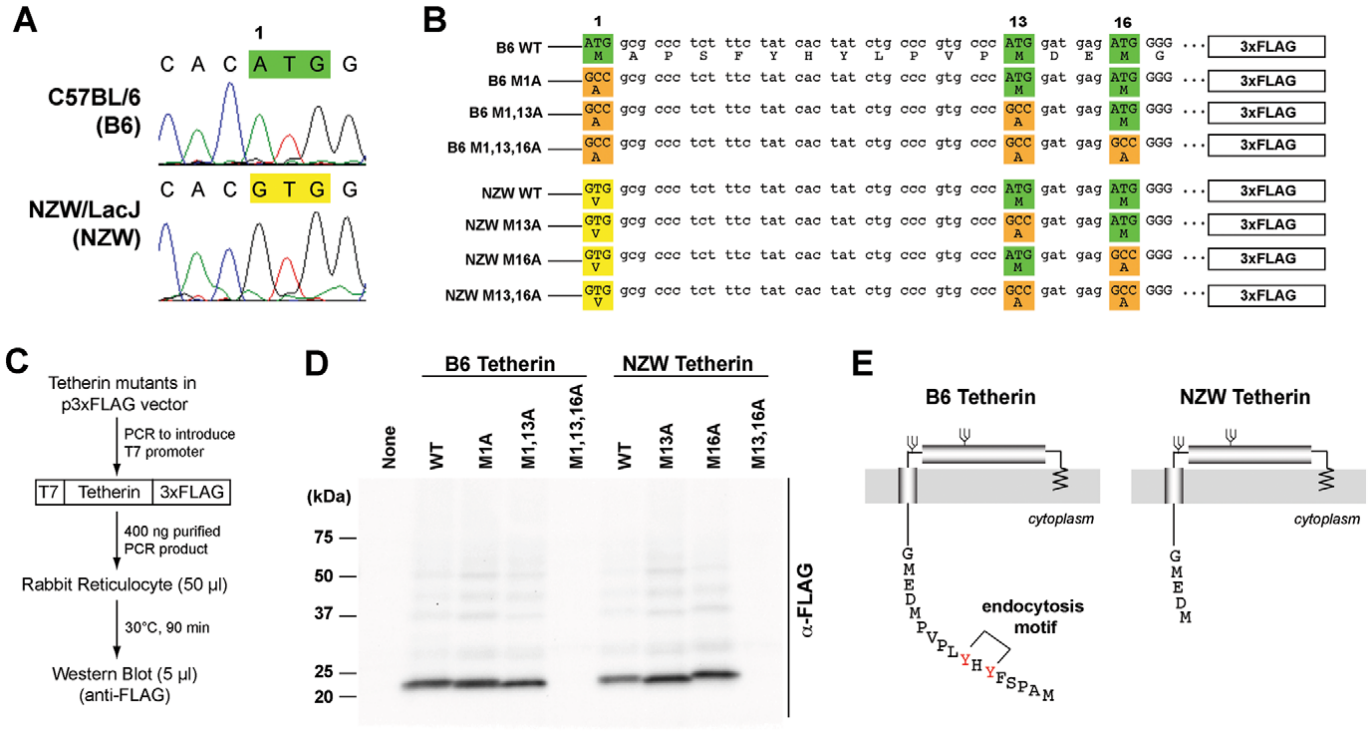
A *Tetherin* SNP in NZW mice results in truncation of the N-terminal cytoplasmic domain. (A) Sequence analysis of B6 versus NZW *Tetherin*. The canonical *Tetherin* ATG (Methionine) start site in B6 mice is mutated to GTG (Valine) in NZW mice, as shown by the sequence chromatogram peaks. (B) *Tetherin* ATG mutant panel. *Tetherin* ATG sites were mutated to GCC (Alanine) in various combinations and linked to a C-terminal 3×FLAG tag. The mutant names indicated on the left were used in subsequent figure panels. (C) *In vitro* translation assay. T7-promoter containing PCR amplicon cassettes were subjected to *in vitro* transcription/translation in rabbit reticulocyte lysates and analyzed by Western blot. (D) Translation efficiency of *Tetherin* mutants. Wild-type B6 and NZW Tetherin were translated at equivalent levels. Expression of the B6 M1A and M1,13A mutants suggested that Methionines at positions 13 and 16 could initiate translation. The absence of NZW M13,16A expression indicated that Valine at position 1 in NZW Tetherin could not initiate translation. The slight shifts in molecular weight qualitatively correspond to translation from remaining Methionine start sites. (E) Model of B6 versus NZW Tetherin. Due to translation from downstream Methionines, NZW mice encode a Tetherin protein that lacks the first 12 amino acids that contain the dual-Tyrosine (YxY) endosomal sorting motif. WT, wild-type.

To investigate if mouse *Tetherin* harbored polymorphisms in putative functional domains, we took advantage of catalogued polymorphisms from multiple inbred mouse strains archived in the *Ensembl* database (Figure S1 in Text S1). A mutation from ATG (Methionine) to GTG (Valine) (dbSNP ID: rs51822354) was found in the *Tetherin* start site of NZW/LacJ (NZW) mice. This was confirmed by sequencing the region from NZW genomic DNA, but was not detected in C57BL/6 (B6) (Figure 1A) or the closely related NZB strain (Figure S1C in Text S1).

Since the *Tetherin* single nucleotide polymorphism (SNP) maps to the canonical start site, we first determined the translational efficiencies of B6 versus NZW Tetherin in a cell-free *in vitro* translation assay. B6 and NZW Tetherin were amplified from primary spleen samples and linked to a C-terminal 3×FLAG tag. We hypothesized that since GUG is a highly inefficient translational initiation codon in mammalian cells [21], downstream Methionines at positions 13 and 16 may be used as alternative start sites. To investigate this possibility, Methionines at positions 1, 13 and 16 were mutated singly or in combination to Alanine (GCC) (Figure 1B). T7-promoter containing PCR amplicons were translated in rabbit reticulocyte lysates and the resulting Tetherin translation products were evaluated by Western blot (Figure 1C). As shown in Figure 1D, wild-type B6 and NZW Tetherin were translated to equivalent levels, demonstrating that the *Tetherin* SNP did not affect Tetherin translation levels. In contrast, Alanine substitutions of NZW Tetherin at Methionine positions 13 and 16 (NZW M13,16A mutant) completely abrogated expression (Figure 1D), demonstrating that Valine at position 1 could not be used for translational initiation. Downstream Methionines at positions 13 and 16 likely initiated Tetherin translation since Alanine substitutions of B6 Tetherin at Methionine position 1 (B6 M1A mutant) and Methionine positions 1 and 13 (B6 M1,13A mutant) still resulted in translation (Figure 1D). Translation from downstream Methionines would result in an approximate 1.4 kDa decrease in molecular weight. We observed slight shifts in molecular weight between B6 WT, M1A and M1,13A Tetherin, as well as between NZW WT, M13A and M16A Tetherin (Figure 1D) that corresponded to translation products from remaining start sites. Overall, these findings indicated that NZW Tetherin is translated from downstream Methionines and lacked the N-terminal 12 amino acids (Figure 1E).

### Higher cell surface expression of NZW Tetherin due to the loss of a critical endosomal sorting motif

**Figure 2:**
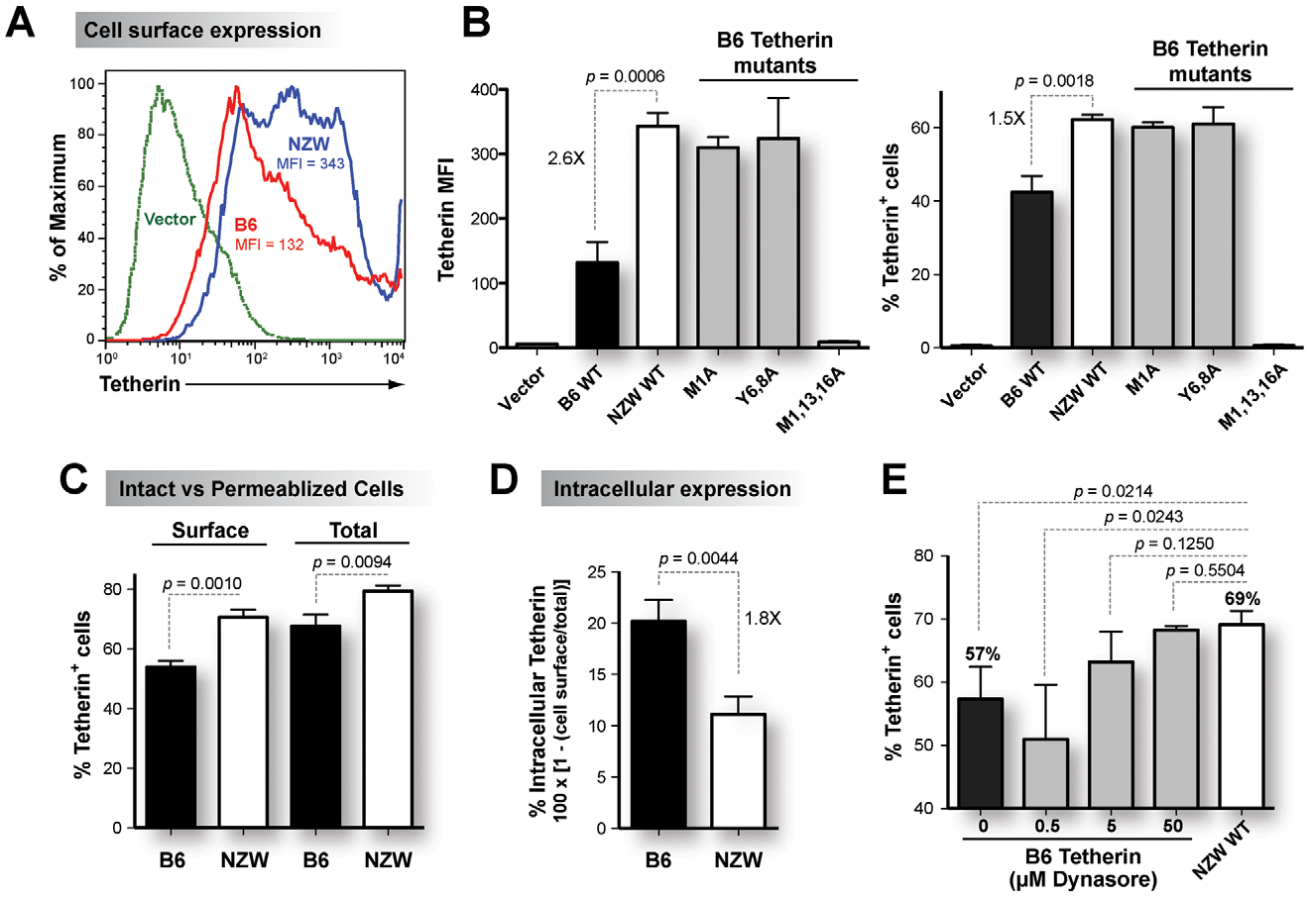
Higher cell surface expression of NZW Tetherin compared to B6 Tetherin *in vitro*. Untagged Tetherin constructs (500 ng) were transfected into 293T cells in triplicate and analyzed after 2 days. (A) Representative flow cytometry histogram profile of B6 versus NZW Tetherin. Higher median fluorescence intensity (MFI) was observed for NZW Tetherin compared to B6 Tetherin. (B) Cell surface expression of Tetherin mutants. Mutating the B6 Tetherin start site (M1A) or endocytosis motif (Y6,8A) to Alanines resulted in similar cell surface expression as NZW Tetherin. Both MFI (left panel) and % Tetherin (right panel) levels are shown. (C) Tetherin expression in intact versus permeabilized cells. In both intact and permeabilized cells, NZW Tetherin was expressed to significantly higher levels than B6 Tetherin. However, % Tetherin expression was higher in permeabilized compared to intact cells, suggesting that a proportion of Tetherin is located intracellularly. (D) Intracellular Tetherin. The percentages of B6 and NZW Tetherin found intracellularly were calculated based on 1 minus the ratio of Tetherin levels in intact (cell surface) versus permeabilized (total) 293T cells multiplied by 100. A higher proportion of B6 Tetherin is expressed intracellularly, consistent with more efficient endocytosis. Inhibition of Tetherin endocytosis *in vitro*. (E) Treatment of B6 Tetherin transfected cells with an endocytosis inhibitor, Dynasore, resulted in similar cell surface levels as NZW Tetherin. Mean values from triplicate measurements, standard deviations as error bars and *p* values from a 2-tailed Student's *t*-test are shown.

The N-terminal cytoplasmic domain of mammalian Tetherins encodes a conserved dual-Tyrosine motif at amino acid positions 6 and 8 that is critical for clathrin-mediated endocytosis [22]–[23]. Substituting Tyrosines at positions 6 and 8 of Tetherin with Alanines (Y6,8A mutant) decreased the internalization of Tetherin thereby increasing Tetherin cell surface expression [22]–[24]. Thus, deletion of the N-terminal 12 amino acids of B6 Tetherin as predicted for NZW Tetherin (Figure 1D) should increase cell surface expression. To test this hypothesis, untagged B6 and NZW Tetherin constructs were transfected into 293T cells and cell surface expression was analyzed. Using immunofluorescence microscopy, we observed brighter and more defined signals for NZW Tetherin on the plasma membrane compared to B6 Tetherin (Figure S2 in Text S1). To quantify cell surface expression, we performed flow cytometry (Figure 2A), measuring both median fluorescence intensity (MFI) and percentage of Tetherin^+^ cells. The percentage of Tetherin^+^ cells was gated based on a FI cut-off that yielded <1% positivity from cells transfected with empty vector. As expected, NZW Tetherin was expressed to a significantly higher level on the surface of transfected 293T cells compared to B6 Tetherin (Figure 2A). In fact, NZW Tetherin was expressed to a similar extent as the B6 Tetherin endocytosis mutant Y6,8A and a B6 Tetherin mutant with the canonical start site mutated to Alanine (M1A) (Figure 2B).

We next compared the total and cell surface levels of B6 and NZW Tetherin by staining transfected 293T cells in an intact (cell surface only) versus permeabilized (cell surface plus intracellular) state. For the same transfected cells, the percentage of Tetherin^+^ cells was higher in permeabilized versus intact cells (Figure 2C), suggesting that a proportion of Tetherin is found inside the cells. Using the ratio of surface versus total Tetherin expression to compute intracellular levels, a significantly higher percentage of B6 Tetherin was found intracellularly compared to NZW Tetherin (Figure 2D) and the B6 M1A and B6 Y6,8A mutants (Figure S3 in Text S1), consistent with more efficient endocytosis of B6 Tetherin. Since the total and cell surface Tetherin expression was higher in NZW Tetherin compared to B6 Tetherin (Figure 2C) despite no difference in translation efficiencies (Figure 1D), we hypothesized that B6 Tetherin may be more efficiently shuttled into endosomal compartments for degradation, as suggested by other reports [24]–[25]. We therefore treated B6 Tetherin-transfected cells with increasing doses of a dynamin-dependent endocytosis inhibitor, Dynasore [26]. Dynasore treatment restored B6 Tetherin cell surface expression to similar levels as NZW Tetherin (Figure 2E; Figure S4 in Text S1). To complement these results, we also utilized a dominant-negative dynamin mutant, K44A, that was previously shown to block an intermediate stage in coated vesicle formation likely due to decreased guanine-nucleotide binding affinity [27]. Co-expression of B6 Tetherin with Dynamin K44A [28] increased B6 Tetherin cell surface expression to the same level as NZW Tetherin (Figure S5 in Text S1). Thus, NZW Tetherin was expressed at higher levels on the cell surface consistent with deletion of the N-terminal 12 amino acid domain and the consequent defect in endosomal recycling.

### NZW Tetherin more potently inhibited retrovirus release than B6 Tetherin *in vitro*

In cell culture, Tetherin activity results in aggregation of retrovirus particles on the infected cell surface, resulting in decreased virion release in the surrounding media [1]–[2], [4]–[5]. Tethering of murine retroviruses by mouse Tetherin has yet to be visualized, but this is highly likely based on the ability of mouse Tetherin to tether HIV-1 as observed by electron microscopy [5] and inhibit the release of Moloney Murine Leukemia Virus [29]. We therefore compared the ability of B6 versus NZW Tetherin to inhibit the release of Friend Murine Leukemia Virus (F-MuLV).

**Figure 3:**
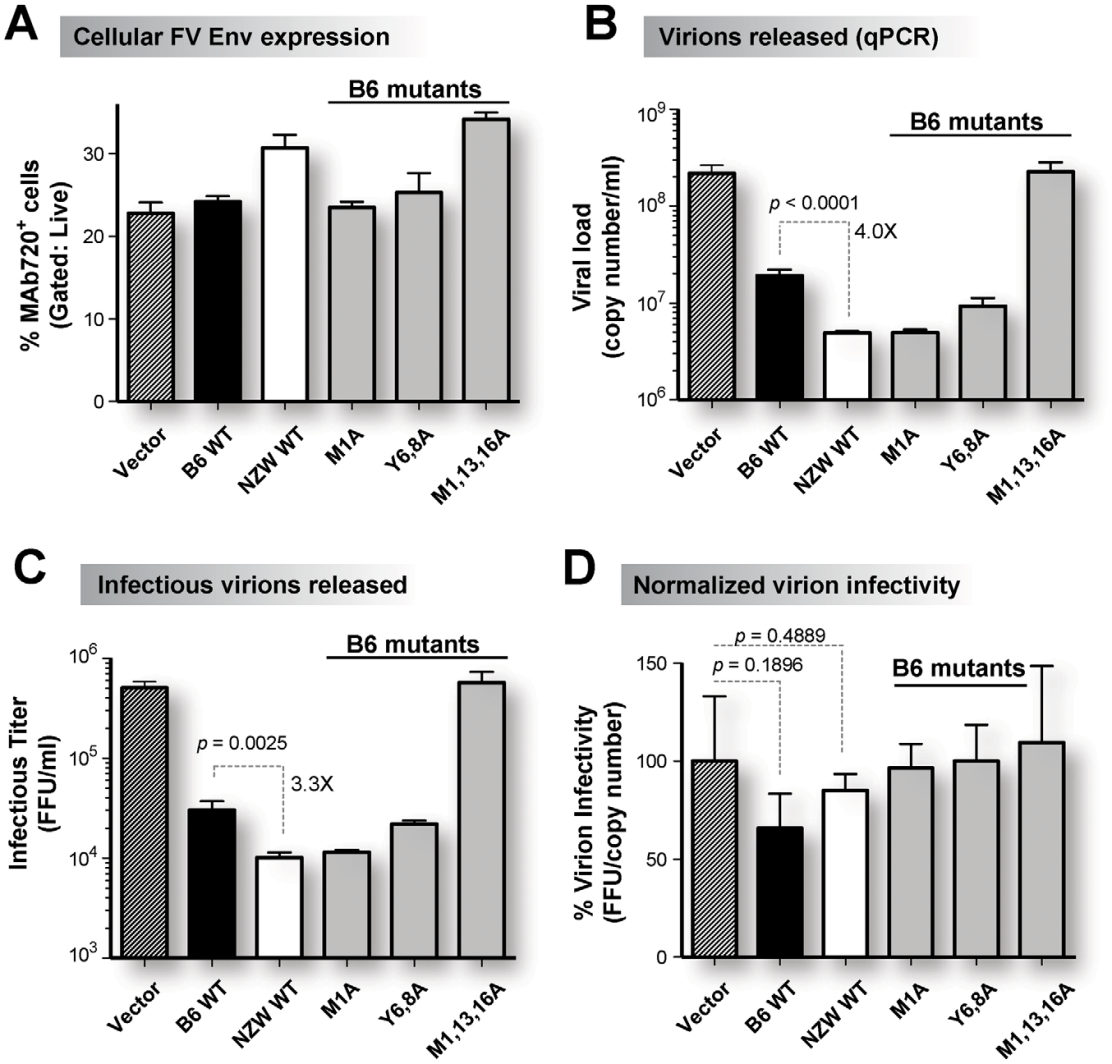
Higher inhibition of virion release by NZW Tetherin versus B6 Tetherin *in vitro*. (A) Cellular FV infection levels. Co-transfection of an infectious F-MuLV molecular clone (1 µg) and Tetherin constructs (50 ng) revealed significant cellular FV envelope gp70 expression by flow cytometry of permeabilized cells following staining with MAb 720. (B) Total virion release measured by quantitative PCR. NZW Tetherin, as well high-surface expression mutants of B6 Tetherin, more efficiently inhibited total virion release compared to B6 Tetherin *in vitro*. (C) Infectious viremia measured by a focal infectivity assay in *Mus dunni* cells. NZW Tetherin, as well as high-surface expression mutants of B6 Tetherin, more efficiently inhibited infectious virion release compared to B6 Tetherin *in vitro*. (D) Virion infectivity. The ratio of infectious titer and viral load was computed and normalized to vector control. There was a moderate reduction in virion infectivity in some Tetherin co-transfected cells but these differences did not reach statistical significance. Mean values from triplicate measurements, standard deviations as error bars and *p* values from a 2-tailed Student's *t*-test are shown.

An F-MuLV molecular clone was co-transfected with untagged mouse Tetherin constructs into 293T cells and the levels of total and infectious virions released after 2 days were measured by quantitative PCR and focal infectivity assays, respectively [30]–[31]. Between 23 to 34% of co-transfected 293T cells expressed FV envelope gp70 based on FACS staining following permeabilization (Figure 3A). Relative to vector control, NZW Tetherin inhibited total virion release 44-fold, while B6 Tetherin inhibited 11-fold (Figure 3B). Infectious virion release was inhibited 50-fold for NZW Tetherin and 17-fold for B6 Tetherin (Figure 3C). The 3 to 4-fold difference in B6 versus NZW Tetherin virion release were statistically-significant (Figure 3B–C). Moreover, the B6 M1A and B6 Y6,8A mutants, which exhibited higher cell surface expression (Figure 2B), also inhibited F-MuLV virion release better than wild type B6 Tetherin (Figure 3B–C). Since Tetherin has been reported to decrease HIV-1 virion infectivity [32], we estimated the infectivity of released F-MuLV virions by calculating the ratio of non-log transformed infectious titers and viral loads, and normalized to vector controls [31]. A moderate reduction in virion infectivity was observed with Tetherin co-transfection but did not reach statistical significance (Figure 3D). Overall, our results demonstrated that NZW Tetherin, which is retained on the cell surface to a greater degree than B6 Tetherin, was a more potent inhibitor of F-MuLV virion release than B6 Tetherin *in vitro*.

### Increased Tetherin cell-surface expression in NZW mice is a recessive trait *in vivo*

**Figure 4:**
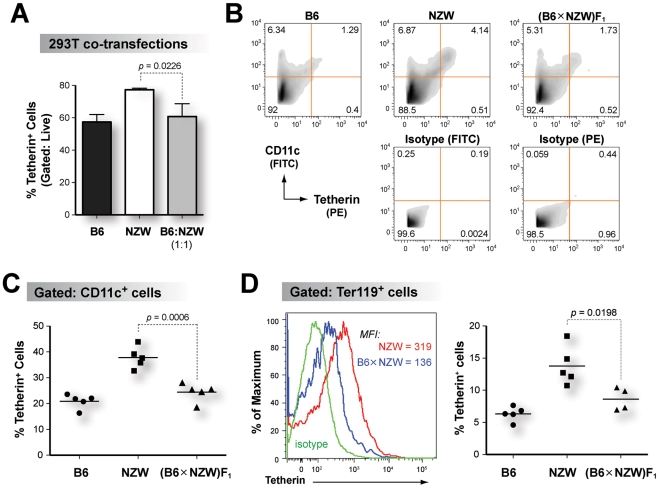
B6 Tetherin cell surface expression is dominant over NZW Tetherin *in vivo*. (A) Cell surface expression of putative B6:NZW heterodimers *in vitro*. Equimolar transfection of B6 and NZW Tetherin resulted in similar cell surface expression as B6 Tetherin *in vitro*. (B) FACS gating strategy. Splenocytes from uninfected B6, NZW and (B6×NZW)F_1_ mice were stained with an anti-Tetherin antibody. Representative flow cytometry plots showing isotype controls used for gating are shown. (C) Tetherin expression in CD11c^+^ dendritic cells. (D) Tetherin expression in Ter119^+^ erythroblasts. Left panel, Tetherin FACS histogram from gated Ter119^+^ cells. Right panel, percentage of Tetherin^+^ cells. In panels C and D, hybrid (B6×NZW)F_1_ mice had significantly lower Tetherin cell surface expression compared to NZW mice. Each dot corresponds to one mouse analyzed. Differences between means were evaluated using a 2-tailed Student's *t*-test.

Previous studies have shown that Tetherin dimers are the functional units critical for restricting virion release *in vitro* [4], [33]. Thus, if B6 and NZW Tetherin heterodimers are formed, the endocytosis motif in B6 Tetherin could lead to decreased cell surface expression of the heterodimer. In support of this notion, co-transfection of B6 and NZW Tetherin at an equimolar ratio led to cell-surface expression that was similar to B6 Tetherin (Figure 4A; Figure S6 in Text S1). To extend this finding *in vivo*, Tetherin cell surface expression in splenocytes of B6, NZW and (B6×NZW)F_1_ mice was evaluated (Figure 4B). Tetherin was expressed to significantly higher cell surface levels in NZW mice compared to B6 mice on dendritic cells and B cells (Figure 4C and Figure S7A in Text S1) which normally express Tetherin [34]–[35]. In addition, higher cell surface expression levels of Tetherin were observed in erythroblasts of NZW compared to B6 mice (Figure 4D and Figure S7B in Text S1). Importantly, Tetherin cell surface expression in hybrid (B6×NZW)F_1_ mice was similar to that of B6 mice (Figure 4B–D; Figure S7A–B in Text S1), indicating that reduced B6 Tetherin cell surface expression was dominant over NZW Tetherin *in vivo*.

### The recessive *Tetherin* SNP in NZW mice significantly inhibits retroviral infection and pathogenesis *in vivo*

**Figure 5:**
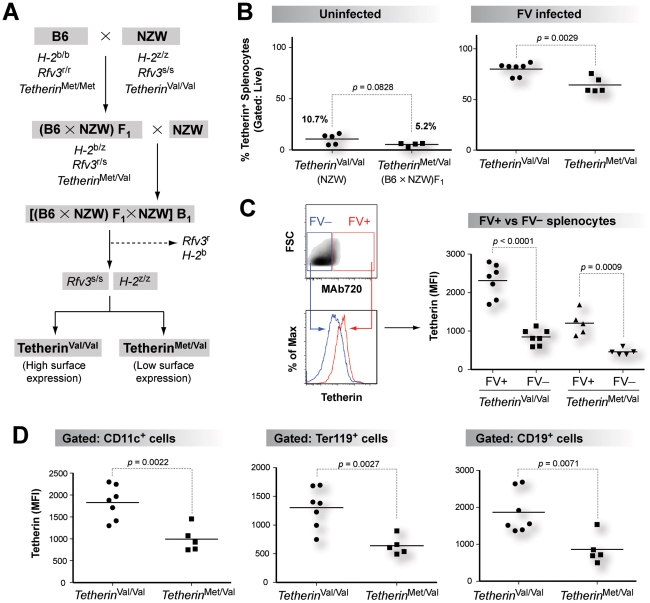
Classical backcrossing strategy to analyze the impact of a *Tetherin* SNP *in vivo*. (A) Mouse backcrossing strategy. Susceptible *Rfv3* ^s/s^ *H2* ^z/z^ mice from a B_1_ backcross were genotyped as *Tetherin* ^Val/Val^ (‘high-Tetherin’ expressors; n = 7) or *Tetherin* ^Met/Val^ (‘low-Tetherin’ expressors; n = 5). The mice were infected with 500 SFFU of NB-tropic FV and biological samples were analyzed at 7 dpi. (B) Induction of Tetherin expression during acute FV infection. The percentage of Tetherin^+^ cells was significantly lower in uninfected (left panel) versus FV infected (right panel) mice. (C) Increased Tetherin expression in FV infected splenocytes. *Left panel*, FV+ versus FV- cells from infected mice were gated based on reactivity to anti-FV envelope gp70 antibody (MAb 720) and Tetherin MFI levels from each gate were compared. *Right panel*, MAb720+ cells showed significantly higher Tetherin MFI levels compared to MAb720- cells. (D) Tetherin MFI levels in FV susceptible cell types. Infected *Tetherin* ^Val/Val^ mice exhibited significantly higher Tetherin expression compared to *Tetherin* ^Met/Val^ mice in CD11c^+^ dendritic cells, Ter119^+^ erythroblasts and CD19^+^ B cells. Each dot corresponds to one mouse analyzed. Differences between means were evaluated using a 2-tailed Student's *t*-test.

The finding that low cell surface expression of B6 Tetherin was dominant over the high cell surface expression of NZW Tetherin provided the opportunity to investigate the effects of Tetherin cell surface expression levels on retroviral pathogenesis. Mice were infected with Friend retrovirus (FV) complex, one of the few retroviruses that cause disease in adult immunocompetent mice, and for which extensive information on the genetics of host resistance and susceptibility has been generated [36] (Table 1). [(B6×NZW)F_1_×NZW]B_1_ offspring were generated, of which half were expected to be *Tetherin* ^Val/Val^ (high-surface expressors) and half *Tetherin* ^Met/Val^ (low-surface expressors) (Figure 5A). The impacts of *Fv1* [37] and *Fv2* [38] host restriction factors (Table 1) were nullified by infecting with a dual (NB)-tropic FV strain and by backcrossing to *Fv2* dominant susceptible NZW mice, respectively. In addition, individual mice were genotyped for *H-2*, which controls T and B cell immunity [36] (Figure S8A in Text S1), and for *Rfv3*, which restricts FV during acute infection, promotes recovery from viremia, influences neutralizing antibody responses and is encoded by *Apobec3* [17], [30] (Figure S8B in Text S1). B_1_ mice (n = 58) were classified into 4 groups based on *H-2* and *Rfv3* genotypes, of which 3 had sufficient sample sizes (≥5 mice per cohort) for FV infection studies (Table S1 in Text S1).

**Table 1:** Known genes that differentially govern acute FV infection in B6 versus NZW mice.

Gene:	B6	NZW	(B6×NZW)F_1_	(F_1_×NZW)	Remarks
Phenotype				B_1_	
***Fv1:***	*b/b*	*n/n*	*n/b*	50% *n/n*	Infection with an NB-tropic strain nullifies the effect of *Fv1* alleles.
				50% *n/b*	
N-tropic FV	resistant	susceptible	resistant		
B-tropic FV	susceptible	resistant	resistant		
NB-tropic FV	susceptible	susceptible	susceptible		
***Fv2:***	*r/r*	*s/s*	*r/s*	100%	*Fv2* ^s^ is dominant. Thus, all B_1_ mice should develop splenomegaly.
				*Fv2* ^s^	
Splenomegaly	No	Yes	Yes		
***Rfv1 (H-2):***	*b/b*	*z/z*	*b/z*	50% *z/z*	CD8 T cell responses, particularly for *H-2* ^b^ mice, are detectable by 6 dpi.
				50% *b/z*	
Cell-mediated Immunity	High	Unknown	Unknown		
***Rfv3 (Apobec3):***	*r/r*	*s/s*	*r/s*	50% *r/s*	*Rfv3* controls recovery from viremia and neutralizing antibody responses. The effects on acute viremia were discovered when *Rfv3* was identified as *Apobec3*.
				50% *s/s*	
Acute Infectious Viremia (7 dpi)	Low	High	Low		

To control for the antiviral effects of *H-2* ^b^ and *Rfv3* ^r^ alleles from the B6 genetic background [17], [39], we initially focused on B_1_ progeny that lacked these resistance alleles (Figure 5A; Table S1 in Text S1). Susceptible *Rfv3* ^s/s^ *H-2* ^z/z^ mice were infected with NB-tropic FV and at 7 days post-infection (dpi), plasma, spleen and bone marrow samples were analyzed. Similar to other viral infections [33], [40], Tetherin expression was highly upregulated following FV infection. Splenocyte Tetherin expression increased from 5-11% Tetherin^+^ splenocytes in uninfected NZW and (B6×NZW)F_1_ mice (Figure 5B, left panel) to 58–87% at 7 dpi (Figure 5B, right panel). In fact, in mice with acute FV infection, FV positive cells showed significantly higher Tetherin cell surface expression compared to FV negative cells (Figure 5C). Interestingly, FV infected *Tetherin* ^Met/Val^ mice still exhibited significantly lower Tetherin cell surface expression in splenic erythroblasts, dendritic cells and B cells as compared to *Tetherin* ^Val/Val^ mice, consistent with their genotypes (Figure 5D).

**Figure 6:**
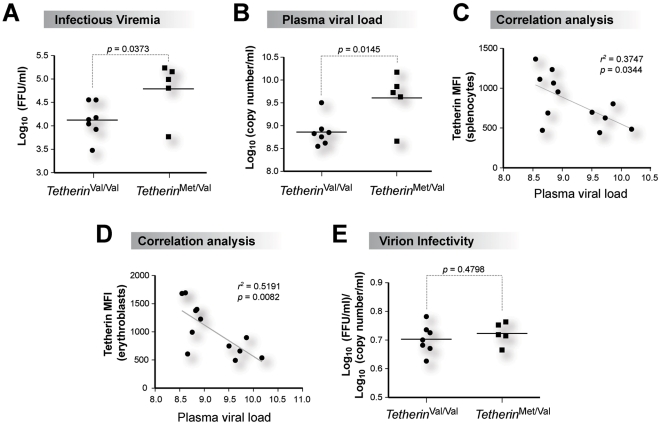
NZW Tetherin inhibits acute FV replication *in vivo*. Susceptible *Rfv3* ^s/s^ *H2* ^z/z^ mice were infected with FV and 7 dpi plasma samples were analyzed against *Tetherin* genotype. (A) Infectious plasma viremia, as measured using a focal infectivity assay. (B) Plasma viral load, as measured by quantitative real-time PCR. In panels A and B, high-Tetherin expressing mice had significantly lower levels of infectious viremia and plasma viral load. (C) Inverse correlation between Tetherin MFI levels in total splenocytes and plasma viral load. (D) Inverse correlation between Tetherin MFI levels in erythroblasts and plasma viral load. In panels C and D, values were obtained using Pearson product-moment correlation analysis. The inverse correlation between plasma viral load and Tetherin erythroblast MFI was more statistically significant than with plasma viral load and Tetherin total splenocyte MFI. (E) Virion infectivity. The ratio of infectious viremia and plasma viral load were compared. No significant differences were obtained. Each dot corresponds to one mouse analyzed. Differences between means were evaluated using a 2-tailed Student's *t*-test.

**Figure 7:**
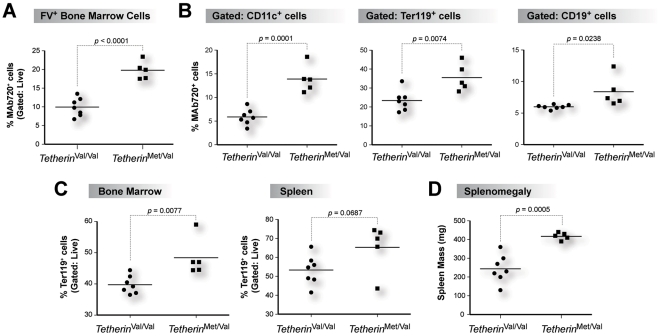
NZW Tetherin inhibits acute cellular FV replication and pathogenesis *in vivo*. (A) Bone marrow FV infection levels. FV infected bone marrow cells were quantified by FACS staining with MAb 720. (B) FV infection of bone marrow cell subsets. MAb 720^+^ cells were quantified on gated CD11c^+^ dendritic cells, Ter119^+^ erythroblasts and CD19^+^ B cells. In panels A and B, high-Tetherin expressing mice showed significantly higher cellular FV infection levels. (C) Erythroblast percentages. The proportions of Ter119^+^ cells from gated live bone marrows (left panel) and spleens (right panel) were measured. FV pathogenesis is characterized by increased erythroblast proliferation. (D) Splenomegaly, based on wet spleen mass. In panels C and D, high-Tetherin expressing mice had significantly lower erythroblast percentages and splenomegaly. Each dot corresponds to an infected mouse. Solid lines correspond to mean values and differences were evaluated using a 2-tailed Student's *t* test.

We next evaluated FV infection levels and pathogenesis determinants at 7 dpi to determine if pathogenesis phenotypes correlated with the *Tetherin* genotype. *Tetherin* ^Met/Val^ low-expressor mice exhibited 5-fold higher infectious plasma viremia (Figure 6A) and 7-fold higher plasma viral loads (Figure 6B) compared to *Tetherin* ^Val/Val^ high-expressor mice. There was a significant inverse correlation between plasma viral load and Tetherin cell surface expression in splenocytes (Figure 6C), but most significantly with erythroblast Tetherin MFI levels (Figure 6D). Virion infectivity, computed from the ratio of infectious viremia and plasma viral load [31], was not significantly different between the two cohorts (Figure 6E). Importantly, *Tetherin* ^Met/Val^ mice had 2-fold higher levels of FV infection in bone marrow cells (Figure 7A) that included dendritic cells, erythroblasts and B cells (Figure 7B). FV-induced proliferation of Ter119^+^ erythroblasts, which eventually leads to splenomegaly and erythroleukemia [37], was also increased in both the bone marrows and spleens of *Tetherin* ^Met/Val^ mice (Figure 7C). In fact, *Tetherin* ^Met/Val^ mice had 2-fold higher levels of splenomegaly compared to *Tetherin* ^Val/Val^ mice (Figure 7D). These findings demonstrate that the recessive NZW Tetherin allele significantly inhibited acute FV replication and pathogenesis *in vivo*.

### NZW *Tetherin* does not impact retrovirus pathogenesis in the context of *Rfv3* resistance

**Figure 8:**
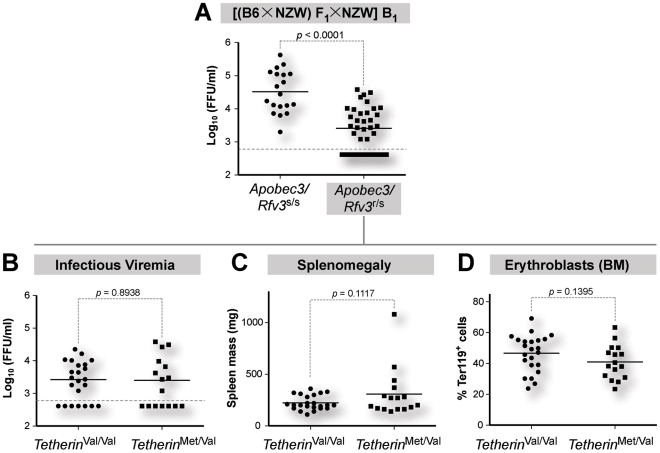
B6 *Apobec3* is a dominant over NZW *Tetherin* restriction *in vivo*. (A) *Apobec3/Rfv3* genotype influence acute infectious viremia. Infectious viremia, as measured using the *Mus dunni* focal infectivity assay, was measured for the entire B_1_ cohort (n = 58). *Apobec3/Rfv3* ^r/s^ mice exhibited significantly lower infectious viremia. *Apobec3/Rfv3* ^r/s^ mice (n = 39) were genotyped as *Tetherin* ^Val/Val^ versus *Tetherin* ^Met/Val^ and various FV infection and pathogenesis parameters were compared. (B) Infectious viremia, split by *Tetherin* genotype from panel A. (C) Splenomegaly, based on wet spleen mass. (D) Bone marrow erythroblast percentages, quantified following FACS staining with Ter119 marker. In panels B to D, *Tetherin* ^Val/Val^ and *Tetherin* ^Met/Val^ exhibited similar levels of infectious viremia, spleen mass and bone marrow erythroblast percentages. Each dot corresponds to an infected mouse. Solid lines correspond to mean values and differences were evaluated using a 2-tailed Student's *t* test.

Several innate retrovirus restriction factors have been identified in the last decade, but whether these factors operate in a synergistic manner to inhibit retroviruses *in vivo* remain unknown. The classical backcrossing approach provided an opportunity to investigate Tetherin restriction in the context of Apobec3/*Rfv3*, a deoxycytidine deaminase that inhibits infectious viremia during acute FV infection [17], [31]. B6 mice are *Rfv3* resistant and express higher levels of *Apobec3* compared to *Rfv3* susceptible NZW mice [41]. Consistent with the dominance of the *Rfv3* resistance allele, B_1_ mice genotyped as *Apobec3/Rfv3* ^r/s^ had significantly lower infectious viremia at 7 dpi compared to *Apobec3/Rfv3* ^s/s^ mice (Figure 8A). *Apobec3/Rfv3* ^r/s^ mice were further genotyped as *Tetherin* ^Met/Val^ or *Tetherin* ^Val/Val^, and infectious viremia (Figure 8B), splenomegaly (Figure 8C) and bone marrow erythroblast levels (Figure 8D) were compared. None of these parameters were significantly different between *Tetherin* ^Met/Val^ versus *Tetherin* ^Val/Val^ mice (Figure 8B–D). The *Tetherin* SNP also did not influence infectious viremia, splenomegaly and bone marrow erythroblast levels when *Apobec3/Rfv3* ^r/s^ mice were analyzed according to their *H-2* haplotypes (Table S1 in Text S1). Thus, the protective effect of NZW Tetherin during acute FV infection was not observed in the context of *Apobec3/Rfv3* resistance.

## Discussion

Despite major progress in elucidating how Tetherin interacts with retroviral pathogens *in vitro*, the demonstration of Tetherin as a potent innate restriction factor *in vivo* remains controversial. In fact, several studies suggested that Tetherin could facilitate cell-to-cell virus spread [8]–[10]. In this study, we identified a *Tetherin* SNP in NZW mice that significantly enhanced Tetherin cell-surface expression, providing the first significant association between *Tetherin* genomic variation and innate retrovirus restriction *in vivo*.

Interrogating the role of the *Tetherin* SNP is not feasible with a straightforward knock-out mouse approach. We therefore utilized a classical genetic backcrossing approach that takes into account the major resistance and susceptibility genes mapped in the FV model [36] and the dominance of the B6 Tetherin cell surface expression phenotype. Our results revealed a direct correlation between *Tetherin* genotype, phenotype, and retroviral restriction. These backcross results excluded contributions by any gene not closely linked to *Tetherin*. While genes tightly linked to *Tetherin* may still contribute to the resistance phenotype, we argue that this is unlikely based on several lines of evidence. First of all, the *in vivo* restriction phenotype was consistent with the *in vitro* phenotype of NZW Tetherin more potently inhibiting FV virion release compared to B6 Tetherin. Furthermore, there was a strong correlation between Tetherin cell surface expression levels mediated by the *Tetherin* SNP and FV replication *in vivo*. Finally, none of the genes flanking *Tetherin* have previously been identified as retrovirus restriction factors (Figure S1A in Text S1). It is most likely that the phenotypes observed were mediated primarily by the *Tetherin* SNP.

While this manuscript was being prepared, a study using *Tetherin* deficient B6 mice revealed that *Tetherin* acts as a resistance gene to counteract Moloney MLV and a pathogenic MLV strain known as LP-BM5 *in vivo* [13]. In Moloney MLV infection of newborn mice, treatment with poly(I:C), a potent inducer of IFN-α, was required to unmask the antiretroviral activity of Tetherin. On the other hand, the impact of *Tetherin* was not observed until after 8 weeks following pathogenic LP-BM5 infection, a timepoint when IFN-α was induced, but fits within the chronic stage of infection and adaptive immune responses. Our results in the FV model were obtained during acute FV infection (7 dpi) and did not require exogenous administration of poly(I:C). Interestingly, the FV stock used in this study, as well as studies that identified the *Fv1, Fv2, Rfv1* and *Rfv3* restriction genes and their molecular counterparts [36], contained Lactate Dehydrogenase-elevating virus (LDV) [42], a potent inducer of Type I IFNs [43]–[44]. Thus, our findings are consistent with the notion that Type I IFN induction is necessary for Tetherin antiretroviral activity *in vivo*. However, our findings differ from [13] in that Type I IFN induction was not sufficient to reveal the antiviral effect of Tetherin. Even with LDV co-infection (and by inference, Type I IFN induction [43]–[44]), the protective effect of NZW Tetherin was not observed in the context of B6 Apobec3/*Rfv3* resistance. Thus, Apobec3 is dominant and not additive with Tetherin restriction *in vivo*. We hypothesize that in addition to the lack of Type I IFN induction, the presence of a potent B6 *Apobec3* gene could explain the weak antiviral effect of B6 *Tetherin* observed in acute Moloney MLV and LP-BM5 infections [13]. Follow-up studies on *Tetherin* retrovirus restriction will likely be more informative in *Rfv3* susceptible genetic backgrounds.

We speculate that the lack of synergy between B6 Apobec3 and NZW Tetherin could be due to mechanistic incompatibility: Apobec3 activity results in the release of non-infectious virions [31], while Tetherin activity prevents virion release [1]–[2]. This model mirrors restriction factor hierarchies that have been observed in the FV model. For example, *Fv1* is dominant over *Fv2* (a B-tropic virus will not efficiently infect *Fv2* susceptible mice that are *Fv1* ^n/n^), while *Fv2* is dominant over *Rfv3* (*Fv2* resistant B6 mice lacking *Apobec3* are expected to recover). The current study suggests the existence of pathways that cross-regulate Apobec3 and Tetherin during acute retrovirus infection *in vivo*.

The finding that NZW Tetherin is a more potent inhibitor of FV infection than B6 Tetherin suggests that high cell surface expression is a key parameter for Tetherin retroviral restriction *in vivo*. This result may be unexpected if Tetherin is viewed primarily as a restriction factor since NZW Tetherin has lost the YxY endocytosis motif that is present in most mammalian Tetherins [20]. Thus, from an evolutionary standpoint, Tetherins with a functional YxY motif should be a more effective configuration for retroviral restriction *in vivo* if this restriction is its primary function. However, Tetherin (or PDCA-1) is a marker of plasmacytoid dendritic cells (pDC) and may have biological functions independent of retroviral restriction, such as the transport of cytokines [34] and regulating the Type I interferon response [45]. Thus, the Tetherin endocytosis motif may have been conserved for these physiological functions and retroviral restriction is part of a dual function. Surprisingly, *Tetherin* deficient mice did not harbor perturbations in pDC function [13]. Further analysis of congenic mice with a canonical (B6), NZW and null Tetherin may yet uncover critical physiological roles for Tetherin *in vivo*.

Studies implicating Tetherin as a modulator of cell-to-cell spread [8]–[10] predict that the higher cell-surface expression of NZW Tetherin should enhance virological synapse formation, retroviral cellular spread and pathogenesis. Moreover, a study demonstrating that Tetherin shuttles virions into endosomal compartments for degradation [25] predict that NZW Tetherin should have weaker antiretroviral activity. Reconciling these studies with our seemingly contradictory results will require tracking the fate of Tetherin-restricted FV virions in congenic mice encoding B6 versus NZW Tetherin. However, we note that most studies on Tetherin were performed *in vitro*, under conditions that lack immune mediators that are present *in vivo*. Aggregation of protein antigens has been known to improve vaccine immunogenicity [46], and Tetherin mediated aggregation of viral particles may confer a similar effect. The ability of a retrovirus restriction factor to promote immune responses is not unprecedented: noninfectious particle release due to B6 Apobec3 activity primed a more effective IgG response directed against intact virions [31]. Future investigations should therefore determine whether cells expressing surface-tethered virion aggregates are more efficiently targeted for innate immune killing and whether NZW Tetherin activity could prime a more effective adaptive immune response.

## Materials and Methods

### Mice strains

C57BL/6J (B6), BALB/cJ and NZW/LacJ mice were purchased from the Jackson Laboratory. All mice were handled in strict accordance with the recommendations in the Guide for the Care and Use of Laboratory Animals of the National Institutes of Health. The protocol was approved by the University of Colorado Health Sciences Center Animal Care and Use Committee [Permit Number B-89709(10)1E]. All infections were performed under isoflurane anaesthesia, and all efforts were made to minimize suffering.

### Cell culture

293T and *Mus dunni* cells were cultured in DMEM (Mediatech) containing 10% Fetal Bovine Serum (Gemini) and penicillin/streptomycin/glutamine (Mediatech).

### Construction of mouse *Tetherin* mutants

Total RNA from B6 and NZW spleens were extracted using the RNAEasy kit (Qiagen) and cDNAs were synthesized using random hexamers in the RT^2^ EZ First Strand synthesis kit (SA Biosciences). Mouse *Tetherin* was amplified using a forward primer 52 bp upstream of the canonical start codon (5′- AAGCTT*GCGGCCGC*TAAGGGCGTGGCCTGGAAAGGGT) and a reverse primer that excludes the stop codon (5′-GAATCC*TCTAGA*AAAGAGCAGGAACAGTGACACT). Cloning sites for *Not*I and *Xba*I (italicized) were included in the primer, allowing for direct subcloning of the PCR amplicon into the p3×FLAG-CMV-14 vector (Sigma). Various Alanine (GCC) mutations were introduced using the Quikchange XL Mutagenesis Kit (Stratagene). A stop codon (TAG) between the last amino acid in *Tetherin* and the 3×FLAG tag was introduced to prepare untagged versions of these constructs. All constructs were verified by DNA sequencing.

### Cell-free *in vitro* translation

T7 expression cassettes were generated from the Tetherin constructs by PCR amplification with a forward primer containing the underlined T7 promoter (5′- TAATACGACTCACTATAGGGTAAGGGCGTGGCCTGGAAAGGGT) and a reverse primer that flanks the 3×FLAG tag. Purified PCR products (400 ng) were added into 50 µl of rabbit reticulocyte lysate from the TNT T7 coupled *in vitro* transcription/translation kit (Promega) and incubated at 30°C for 90 min. 5 µl of lysate was solubilized in 50 µl of Laemmli buffer, and 5 µl of solubilized lysate was loaded in a 4–20% gradient Criterion Gel (Biorad) and transferred into PVDF membranes (Invitrogen). High levels of reticulocyte proteins below 20 kDa were cut out of the membrane prior to incubation with antibodies. Membranes were blocked with 5% skim milk for 2 h, probed with 1∶500 dilution of anti-FLAG M2 monoclonal antibody (Sigma) overnight, washed and incubated with 1∶5000 anti-mouse horseradish peroxidase conjugate (Amersham) for 1 h. The blots were developed using the Western Lightning reagent (Perkin-Elmer) and visualized in a Chemidoc XRS+ system (Biorad). Commercial anti-Tetherin antibodies from Abnova (catalogue # PAB13047) and Abcam (catalogue # ab14694) did not detect mouse Tetherin by Western blot analysis (data not shown).

### Cell surface expression of transfected constructs

Untagged Tetherin expression plasmids (500 ng) were transfected in triplicate into individual wells of a 6-well plate containing 80,000 293T cells using the Fugene 6 reagent (Promega). After 2 days, the cells were washed twice and harvested with 1 ml of FACS buffer (PBS+2% FBS). Different doses of Dynasore (Sigma) were added 4 hr before harvest in some experiments. To quantify total Tetherin expression (cell surface plus intracellular), 293T cells were permeabilized and stained using the Cytofix/Cytoperm Kit (BD Biosciences). As an alternative to Dynasore treatment to block endocytosis, 1 µg of wild-type or K44A mutant dynamin in an eGFP-expression vector (generously provided by P. De Camilli through Addgene, plasmids 22163 and 22197; [28]) and 250 ng of B6 or NZW Tetherin were co-transfected into 293T cells using Fugene 6. Tetherin expression was analyzed in gated dynamin-eGFP+ cells. In all cases, 60 µl of the cell suspension was stained with 0.5 µl of PE-conjugated anti-PDCA-1 (eBioscience; clone eBio927) for 30 min on ice, washed twice with FACS buffer then fixed with 1% paraformaldehyde. Samples were analyzed in a FACSCalibur machine (BD Biosciences), collecting at least 80,000 total events.

### Fluorescence microscopy

Untagged Tetherin constructs as well as vector control (100 ng) were transfected into 293T cells using Fugene 6 in poly-D-Lysine coated chamber slides (BD Biocoat; BD Biosciences). After 2 days, the cells were washed with PBS, fixed with 4% paraformaldehyde then blocked for 1 hr at room temperature with PBS containing 8% normal goat serum and 0.02% Triton X-100 (Sigma). The samples were stained with 1∶100 anti-PDCA1 Alexa-488 (eBioscience) overnight at 4°C. The following day, the cells were stained with diamidino2-phenylindole (DAPI; Sigma) for 10 min at room temperature, and then washed 3× with PBS. The samples were visualized in a Zeiss Axioplan2 microscope.

### F-MuLV *in vitro* restriction assay

The F-MuLV molecular clone pLRB302 [47] (1 µg) was co-transfected in triplicate with 50 ng Tetherin expression constructs into 6-well plates containing 80,000 293T cells plated the previous day with Fugene 6 (Promega). After 2 days, MAb 720 supernatant was used to detect FV envelope gp70 expression by incubating permeabilized cells for 30 min, followed by 2 washes with FACS buffer and incubation for 30 min with goat anti-mouse IgG1 conjugated to APC (Columbia Biosciences). Infectious titers in day 2 supernatants were quantified using a focal infectivity assay in *Mus dunni* cells with MAb 720 [17]. Total virion titers were determined using a quantitative real-time PCR based viral load assay [30]–[31] from 100 µl of culture supernatant treated with 2.5U DNAseI (Benzonase; EMD Millipore) for 30 min at room temperature. The same assay was also used to measure plasma viral load from 50 µl of infected mouse plasma. Quantitative PCR was performed at least in triplicate and had PCR efficiencies >90%.

### *H-2, Apobec3* and *Tetherin* genotyping

*H-2* was genotyped by directly sequencing a 1.0-kb *H2-Q1* PCR fragment amplified using primers H2.F (AACCTGGGTCAGGTCCTTCT) and H2.R (CATGGCTGACAGAGGCTACA) (Figure S8A in Text S1). To genotype *Apobec3/Rfv3*, we used a multiplex primer set consisting of primers spanning (mA3.F and mA3.R) and positioned (LTR.F) within a 530 bp retroviral xenotropic murine leukemia virus (X-MLV) insertion in B6 mice [30], [41] (Figure S8B in Text S1). The primer sequences are: mA3.F (TTCACAACCCCCATACTTGG); mA3.R (CAGGCTGGTCTCAAACGATA); and LTR.F (TTGGGGAACCTGAAACTGAG). To genotype the start codon mutation in *Tetherin*, we amplified a 582 bp fragment spanning the start codon using primers Bst2.F (AAACCTTGGCCTTTGGTCTT) and Bst2.R (TGTGACGGCGAAGTAGATTG) and directly sequenced the PCR products using an internal primer Bst2.seq (GCGGACAGCCACTGTTAAGT) (Figure S1C in Text S1). Tail DNA samples (10 ng) were added into a 20 µl PCR reaction consisting of 1× Phusion HF Buffer, 10 mM dNTP, 20 pmol of primers and 1.25 U of Hot Start Phusion polymerase (New England Biolabs). Cycling conditions in a PE 9700 machine included a 30 s hot-start at 98°C, followed by 30 cycles of 98°C 10 s denaturation, 60°C 30 s annealing and 72°C 1 min elongation.

### FV infection of mice and immunophenotyping

The FV stock used in this study contains an NB-tropic F-MuLV helper virus, a replication-defective Spleen-Focus Forming Virus and LDV. LDV is a natural mouse virus endemic in wild mouse populations, and its presence in the FV stocks has been traced to the 1960s from naturally infected mice [48]–[49]. Major findings that include the identification of *Fv1*, *Fv2*, *Rfv1* and *Rfv3* and their molecular counterparts utilized LDV+ FV stocks [36], [42]. These 4 genes were used to classify and genotype the B_1_ cohort. Thus, an LDV+ FV inoculum was essential for this study. LDV was shown to stimulate Type I IFN production and modulates adaptive immune responses, but does not significantly influence acute FV infection levels [42]. FV inoculum stocks were prepared in BALB/c mice and had equivalent titers in BALB/c and NZW mice (data not shown). NB-tropic FV (500 SFFU) was infected intravenously in 300 µl RPMI and at 7 dpi, plasma samples, bone marrows and spleens were harvested. Due to severe male aggression, infections were performed in female B_1_ mice, without prior knowledge of the *Tetherin* genotype. Bone marrow and spleen cells (10^6^ cells) were stained with MAb 720 for 30 min, then co-stained with: Ter119-FITC (clone TER-119), CD3-Alexa700 (17A2), (BD Biosciences); PDCA-1-PE (eBio927), CD11c-PE-Cy7 (N418), (eBioscience); CD19-PerCP-Cy5.5 (6D5) (Biolegend) and anti-mouse IgG1-APC (Columbia Biosciences). Isotype controls and cells from uninfected mice were used for gating. The multicolor FACS panel yielded similar data from mice spleen and bone marrow with or without the addition of a mouse anti-CD16 Fc blocker (BD Biosciences) (data not shown). Cells were processed in an LSR-II flow cytometer (BD Biosciences), collecting up to 250,000 events per sample. Datasets were analyzed using the Flowjo software (Treestar).

## Supporting Information

Text S1 Supporting Figures S1 to S8 and Supporting Table 1 are presented with the corresponding legends.
(PDF)
