# Supplementary material for: A Single Nucleotide Polymorphism in Tetherin Promotes Retrovirus Restriction In Vivo
Source: PLoS Pathog. 2012 Mar 22;8(3):e1002596. doi: 10.1371/journal.ppat.1002596 (PMC3310811; doi:10.1371/journal.ppat.1002596)
Supplement: Text S1 — Supporting Figures S1 to S8 and Supporting Table 1 are presented with the corresponding legends. (PDF) [file ppat.1002596.s001.pdf]

Text S1. Supporting Information

A Single Nucleotide Polymorphism in *Tetherin* Promotes Retrovirus Restriction *In Vivo*

Bradley S. Barrett[1], Diana S. Smith[1], Sam X. Li[2], Kejun Guo[1], Kim J. Hasenkrug[4], and Mario L. Santiago[1,2,3]†

†To whom correspondence should be addressed. Email: mario.santiago@ucdenver.edu.

This *.pdf file includes:

Supporting Figures S1 to S8
Supporting Table 1

SUPPORTING INFORMATION

Supporting Figure 1. Detection of a *Tetherin* single nucleotide polymorphism (SNP) in NZW/LacJ mice.

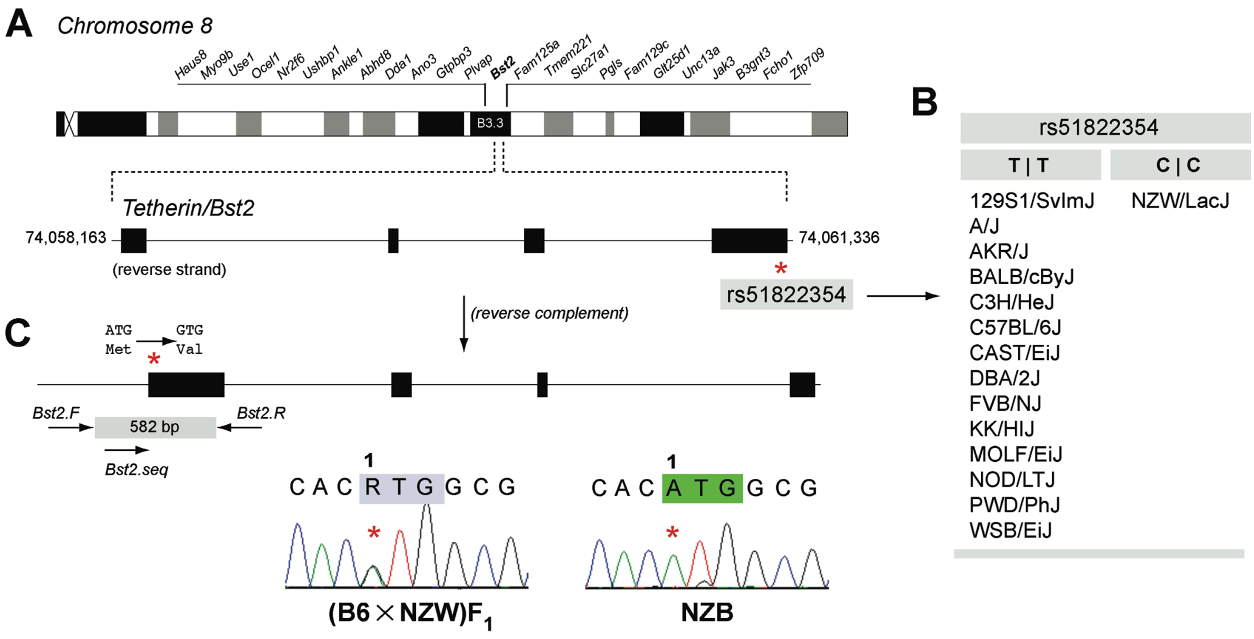

(A) Chromosome location of a novel *Tetherin* SNP. Data mining of mouse genome sequences in the Ensembl site (NCBI m37 assembly) uncovered a SNP in chromosome 8, rs51822354, that maps in the coding region of *Tetherin/Bst2*. (B) The rs51822354 SNP is unique to NZW/LacJ (NZW) mice. Genotypes obtained from PERLEGEN and TWGNF datasets reveal that the C|C genotype is unique to NZW mice. (C) Genotyping approach for the *Tetherin* SNP. The rs51822354 SNP (highlighted in a red asterisk) mutates the Tetherin start codon (Methionine) to a Valine. This was confirmed in NZW mice by direct sequencing of a 582 bp fragment spanning the start codon (Figure 1A). Shown here are representative chromatograms used for genotyping heterozygous (B6 × NZW)F_1 mice. Surprisingly, the related NZB strain does not share the *Tetherin* polymorphism with NZW mice.

Supporting Figure 2. Tetherin expression in 293T cells visualized by fluorescence microscopy.

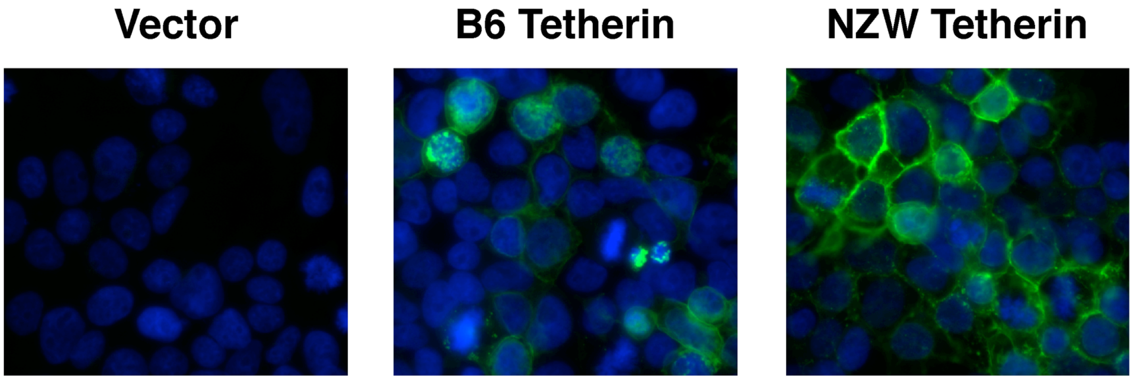

Untagged Tetherin constructs (100 ng) were transfected into 293T cells and stained with an anti-Tetherin antibody directly conjugated to Alexa-488. Tetherin expression is shown in green, while nuclear staining with DAPI is shown in blue. A 40× panel showing several Tetherin-expressing cells are shown. Background staining is shown with vector control (leftmost panel). NZW Tetherin consistently stained brighter and more frequently localized to the plasma membrane compared to B6 Tetherin.

Supporting Figure 3. Intracellular expression of B6 Tetherin endocytosis mutants.

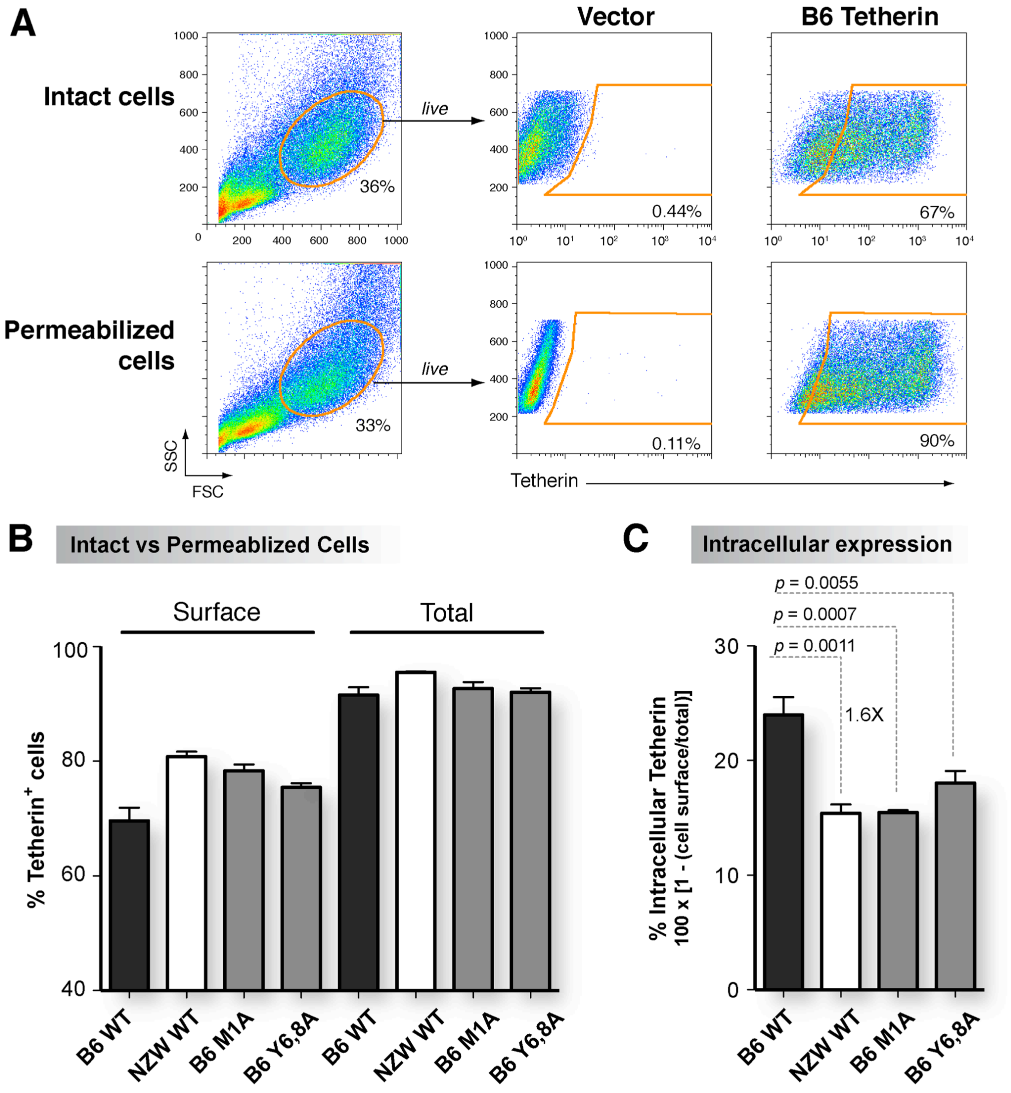

293T cells were transfected with 500 ng untagged Tetherin constructs and analyzed after 2 days. (A) Strategy to quantify Tetherin$^+$ 293T cells in an intact (*upper*) versus permeabilized (*lower*) state. Vector control was used for gating and B6 Tetherin frequencies are shown as an example. (B) Percentage of Tetherin$^+$ cells expressed in intact (surface) versus permeabilized (total) cells. Total Tetherin expression was consistently higher than cell surface expression, suggesting that a proportion of Tetherin is located intracellularly. (C) Intracellular Tetherin levels. The percentage of Tetherin expressed intracellularly was calculated by multiplying 1 minus the ratio of surface versus total Tetherin expression by 100. Data were based on triplicate measurements, error bars correspond to standard deviations and differences between means were calculated using a 2-tailed Student's *t* test.

Supporting Figure 4. Blocking B6 Tetherin endocytosis with Dynasore.

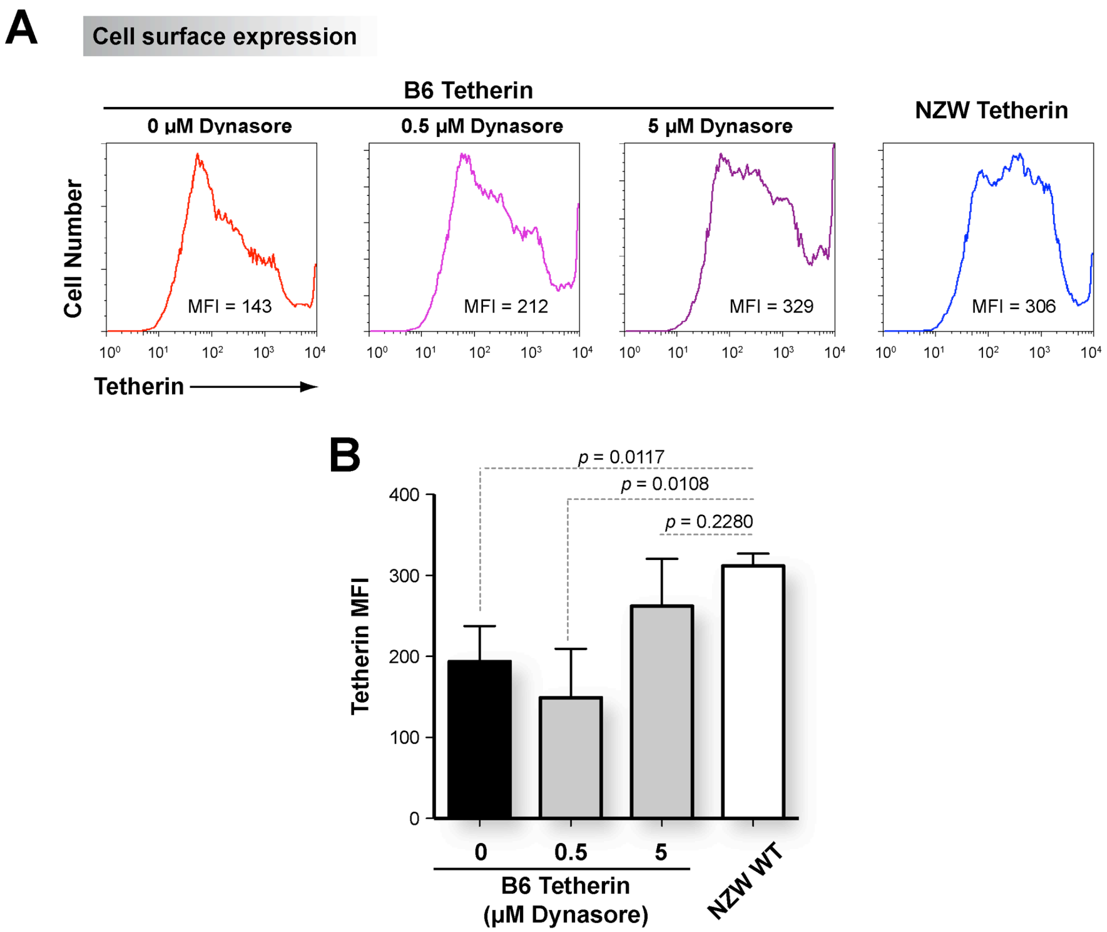

(A) Representative histograms demonstrating that treatment of B6 Tetherin transfected 293T cells with the endocytosis inhibitor Dynasore resulted in increased MFI levels. (B) At 5 µM Dynasore dose, there was no significant difference in Tetherin MFI levels between treated B6 Tetherin and untreated NZW Tetherin. Data were based on triplicate measurements, error bars correspond to standard deviations and differences between means were calculated using a 2-tailed Student's *t* test.

Supporting Figure 5. Blocking B6 Tetherin endocytosis with a dominant-negative dynamin mutant.

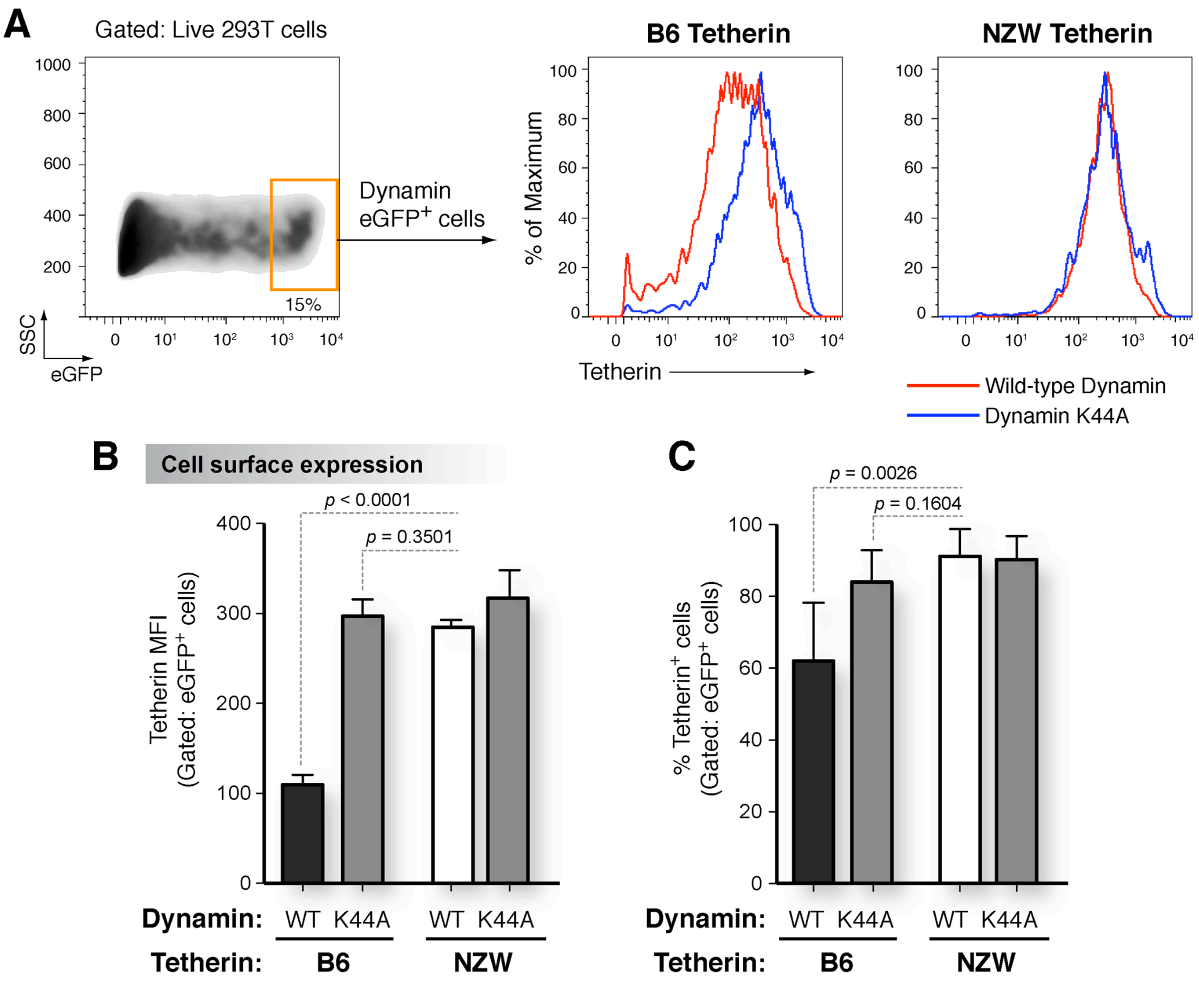

293T cells were co-transfected with untagged Tetherin (250 ng) and either wild-type or K44A Dynamin-eGFP (1 µg) constructs. After 2 days, the cells were subjected to flow cytometry. (A) Gating strategy and Tetherin cell surface expression analysis. Tetherin expression levels were analyzed from gated eGFP+ cells. Right panels correspond to representative histograms, showing increased B6 Tetherin surface levels following co-expression with the dominant-negative K44A dynamin mutant. (B) Increased B6 Tetherin MFI following blockade of endocytosis using a dominant-negative dynamin mutant. (C) Increased percentage of B6 Tetherin expression following blockade of endocytosis using a dominant-negative dynamin mutant. For panels B and C, data were based on triplicate measurements, error bars correspond to standard deviations and differences between means were calculated using a 2-tailed Student's *t* test.

Supporting Figure 6. B6 Tetherin cell surface expression is dominant over NZW Tetherin *in vitro*.

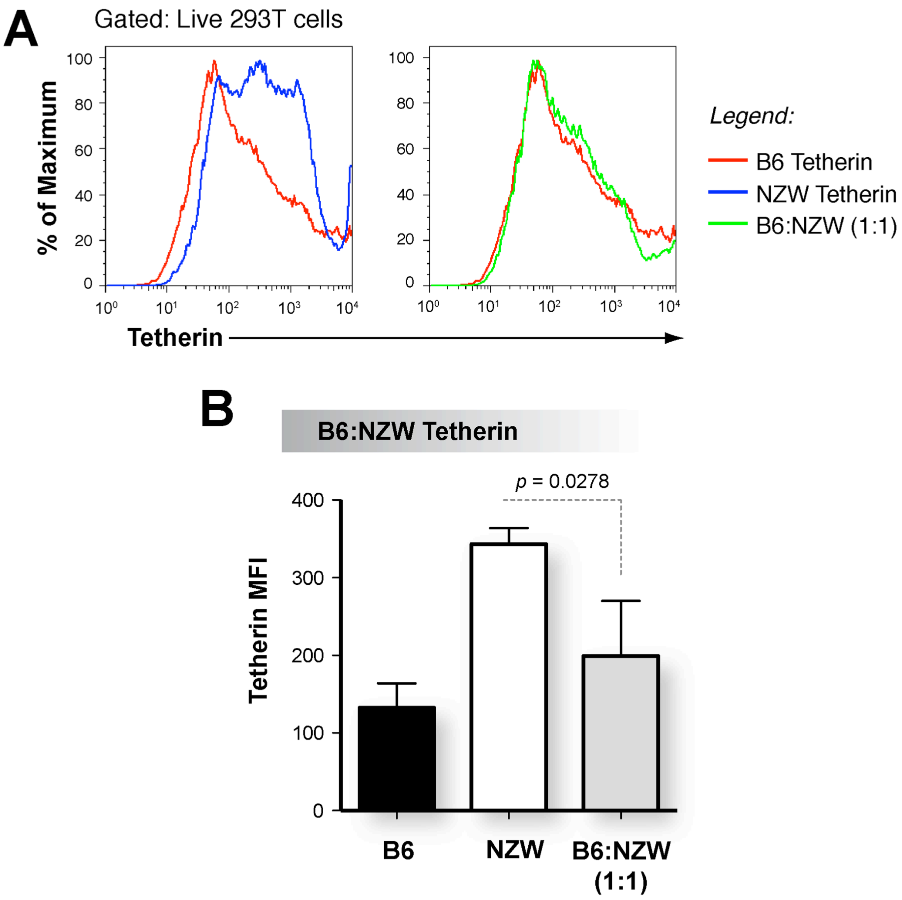

Tetherin MFI levels in 293T cells following equimolar co-transfection of B6 and NZW Tetherin (250 ng each) were compared to 500 ng individual transfections with B6 or NZW Tetherin. (A) Distinct histogram profiles were observed between B6 and NZW Tetherin (*left*), but not when B6 and NZW Tetherin were co-transfected at equimolar levels (*right*). (B) Equimolar B6:NZW co-transfection resulted in decreased Tetherin MFI levels, consistent with the formation of a heterodimer that is endocytosed as efficiently as B6 Tetherin. Data were based on triplicate measurements, error bars correspond to standard deviations and differences between means were calculated using a 2-tailed Student's *t* test.

Supporting Figure 7. Baseline information on B6, NZW and F_1 hybrid mice.

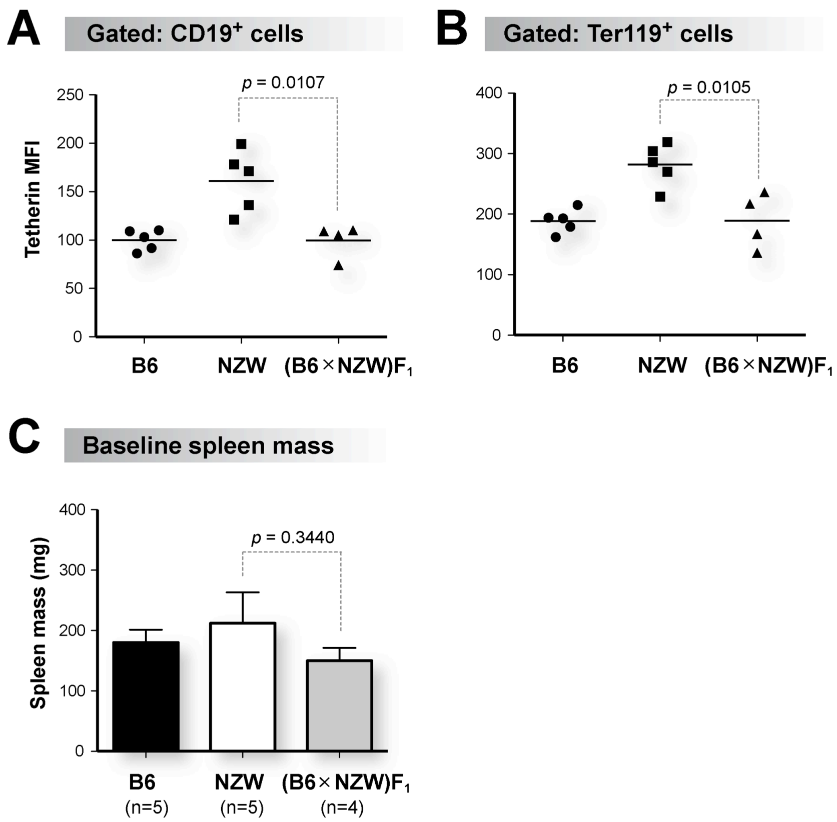

Spleens from uninfected B6, NZW and F_1 hybrid mice were harvested. Tetherin MFI levels in (A) B cells and (B) erythroblasts. Each dot corresponds to a one mouse analyzed. (C) Baseline spleen mass for these three strains were equivalent. Differences between means were calculated using a 2-tailed Student's *t* test.

Supporting Figure 8. Genotyping (B6 × NZW)B$_1$ mice for the *H-2* and *Rfv3* locus.

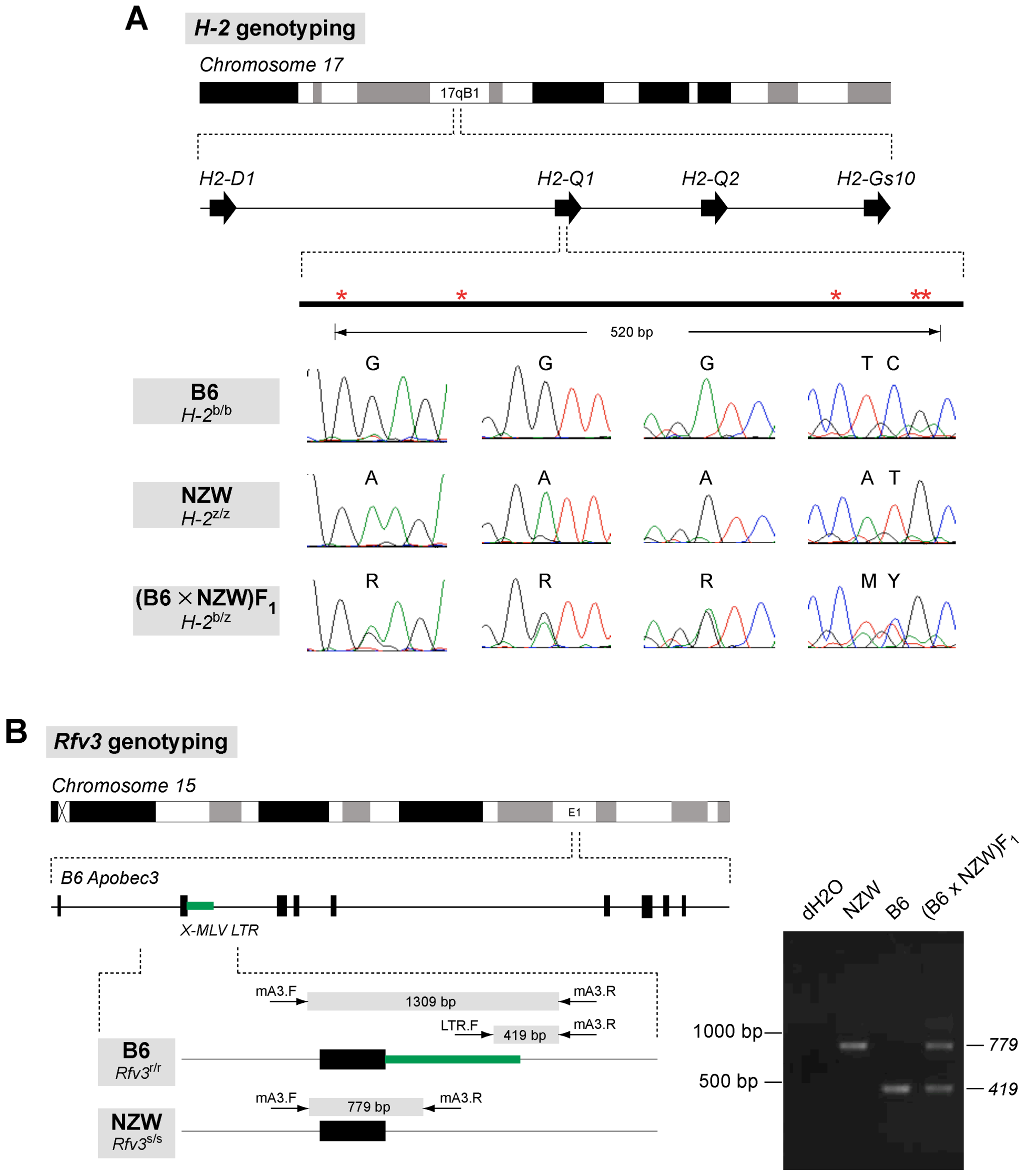

(A) *H-2* genotyping. A 1.0-kb segment of the *H-2* locus (*H2-Q1*) was amplified from the Class I region in chromosome 17. Amplicons were directly sequenced using the reverse primer and 5 SNPs (red asterisks) that vary between B6 and NZW mice within a 520 bp region were utilized for genotyping B$_1$ mice. *H-2*$^{b/z}$ mice will have ambiguous bases at these SNP positions, as exemplified by overlapping chromatogram peaks for (B6 × NZW)F$_1$ mice. (B) *Apobec3/*Rfv3 genotyping. *Rfv3* resistant mice (B6) encode a 530 bp xenotropic murine leukemia virus long terminal repeat (X-MLV LTR) insertion near the *Apobec3* exon 2 splice junction (green line). A PCR genotyping scheme was developed by designing primers (arrows) that flank (mA3.F and mA3.R) and sit (LTR.F) on the X-MLV insertion. Amplification of different size fragments (gray bars) can then be used to distinguish *Rfv3*^{r} (B6) versus *Rfv3*^{s} (NZW) mice. Agarose gel electrophoresis of heterozygous *Rfv3*$^{r/s}$ mice should reveal at least two predominant bands of 419 bp and 779 bp. The 1.3 kb fragment is often not detected in the multiplex PCR, but the lower bands should be sufficient for correct genotyping of B$_1$ mice.

Supporting Table 1. Summary characteristics of (B6 × NZW)B_1 cohort.

	**Genotype**[a]		**Progeny**[b]		**FV Infection Parameters at 7 dpi**[c] (*Tetherin*$^{Val/Val}$ vs. *Tetherin*$^{Met/Val}$)		
**Group**[a]	*H-2*	*Rfv3*	*Tetherin* (Val/Val)	*Tetherin* (Met/Val)	Infectious Viremia	Spleen Size	Ter119$^+$ BM cells
1[d]	*z/z*	*s/s*	7	5	0.0373	0.0005	0.0077
2	*z/z*	*r/s*	15	7	0.7804	0.2606	0.4285
3	*b/z*	*s/s*	3	4	(Insufficient sample size)[e]		
4	*b/z*	*r/s*	8	9	0.5104	0.1142	0.8881

[a]B_1 mice (n=58) segregated into 4 genotype groups based on *H-2* and *Rfv3*.

[b]Number of mice genotyped for the *Tetherin* canonical start site.

[c]*P* values following comparison of *Tetherin*$^{Val/Val}$ versus *Tetherin*$^{Met/Val}$ mice using a 2-tailed Student's *t*-test. *P* values <0.05 were considered statistically significant.

[d]Plasma viral load and cellular FV infection in the BM were also significantly different between *Tetherin*$^{Val/Val}$ and *Tetherin*$^{Met/Val}$ mice for Group 1 (p=0.0145 and p<0.0001, respectively).

[e]No significant difference was observed for each parameter with Group 3, but the sample size may be limited.
